# Emergence of SARS-CoV-2 Omicron sub-lineage JN.1 in Bangladesh

**DOI:** 10.1128/mra.00130-24

**Published:** 2024-04-23

**Authors:** Manjur Hossain Khan Jony, Ahmed Nawsher Alam, Md. Abdullah Omar Nasif, Sharmin Sultana, Rubaid Anwar, Mithun Rudra, Mahbubur Rahman, Mustafizur Rahman, Firdausi Qadri, Tahmina Shirin

**Affiliations:** 1 Institute of Epidemiology, Disease Control and Research (IEDCR), Dhaka, Bangladesh; 2 International Centre for Diarrhoeal Disease Research, Dhaka, Bangladesh; Portland State University, Portland, Oregon, USA

**Keywords:** SARS-CoV-2, Omicron, JN.1, COVID-19, Bangladesh

## Abstract

We report complete genome sequences of 14 severe acute respiratory syndrome coronavirus 2 Omicron sub-lineage JN.1 obtained from Bangladeshi individuals between 19 December 2023 and 21 January 2024. All sequence data were generated by Oxford Nanopore Sequencing Technology using the amplicon sequencing approach developed by the ARTIC network.

## ANNOUNCEMENT

Bangladesh reported the first confirmed case of severe acute respiratory syndrome coronavirus 2 (SARS-CoV-2) on 8 March 2020, and as of 3 February 2024, the country reported 2 million COVID-19 (coronavirus disease 2019) confirmed cases (1). Between October 2022 and May 2023, the circulating strains were Omicron sub-lineages, XBB.1.16, XBB.2.3, FL.4 (alias of XBB.1.9.1.4), and XBB.3 (2). After the emergence and rapid spread of JN.1 in different countries, WHO classified it as a Variant of Interest on 20 December 2023 (3). Institute of Epidemiology, Disease Control and Research (IEDCR), a mandated institute for disease outbreak investigation and response and surveillance in Bangladesh, has been conducting the National Influenza Surveillance, Bangladesh (NISB) since 2010 and National Respiratory Pathogen Genomic Surveillance in Bangladesh since 2022. IEDCR as the national reference laboratory for COVID-19 routinely collects samples (door-to-door, booth collection, and referred samples from suspected cases from different hospitals all over the country) for COVID-19 testing. The IEDCR, functioning as the National Influenza Centre (NIC) also performs Influenza and SARS-CoV-2 multiplex PCR testing from samples from the NISB platform. From 1 December 2023 to 31 January 2024, IEDCR tested a total of 7,228 suspected cases of COVID-19 and detected 93 as positive for SARS-CoV-2. The positive samples (*n* = 14) with *C_T_* value <28 were selected for sequencing. This genomic surveillance is an ongoing activity that was approved by the institutional review board of the IEDCR (protocol IEDCR/IRB/2020/11).

Viral RNA was extracted from nasopharyngeal swab samples using the QIAamp viral RNA Mini Kit (Qiagen, Germany). cDNA library was synthesized using LunaScript RT SuperMix Kit (New England Biolabs, USA), followed by amplification with ARTIC v4.1 primer-based multiplex PCR (4) using Q5 high-fidelity DNA polymerase (New England Biolabs, USA). Amplicons were cleaned up using AMPure Xp Beads (Beckman Coulter, USA), end-repaired with NEBNext Ultra II end repair/dA-tailing module (New England Biolabs, USA), followed by the native barcoding using EXP-NBD104 Kit (Oxford Nanopore Technologies, UK). Libraries were pooled, cleaned with AMPure XP beads (Beckman Coulter, USA), and then sequenced via a FLO-MIN106D flow cell (R9.4.1) for 8 hours. Raw reads were base called and demultiplexed with MinKNOW v22.12.5 integrated with MinION Mk1c. Reads were quality controlled, filtered, and assembled, and variants were called using wf-artic pipeline through EPI2ME Labs (5). All tools were run with default parameters unless otherwise specified.

**Fig 1:**
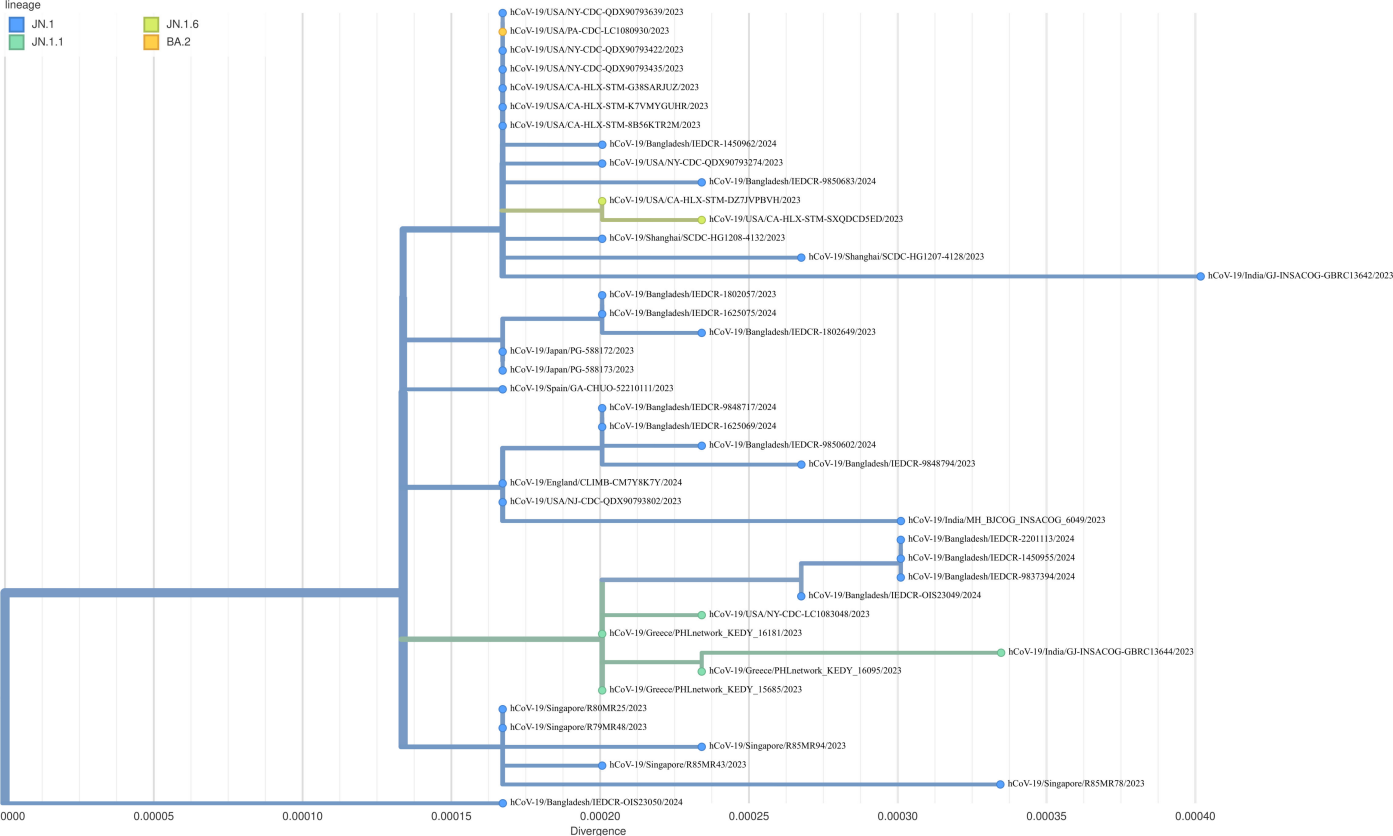
Phylogenetic analysis of coding-complete SARS-CoV-2 Omicron sub-lineage JN.1 genomes. A total of 43 viral genomes are displayed, including study strains. The tree was built and annotated using Augur. Tips are colored according to lineage. The branch length is indicated by divergence.

A total of 2,574,954 reads were obtained (ranging from 69,016 to 430,999 reads per sample), and a substantially complete genome (mean breadth 94.8% to 99.9%) was obtained for each sample. The genomic information is summarized in Table 1. All the sequences are assigned to Clade 23I and PANGO Lineage JN.1 by Nextstrain (www.clades.nextstrain.org). The consensus of 14 sequences and 29 closely related sequences from Global Initiative of Sharing All Influenza Data using the audacity instant (v5.1.0) were aligned using MAFFT v7.4 (6). A phylogenetic tree (Fig. 1) was constructed using Nextstrain tool Augur and visualized in Auspice (www.clades.nextstrain.org). The study sequences formed three separate clusters and were closely related to the strains from Japan, the UK, and the USA.

**TABLE 1:** Data for Bangladesh SARS-CoV-2 Omicron JN.1 sub-lineage (www.gisaid.org)

Sample ID	Date (mo-day-yr) of sample collection	Age (yr)	Sex	Sampling location	Symptoms	GenBank accession no.	GISAID^*a*^ accession no.	SRA^*b*^ accession no.	Number of raw reads	Sequence length (bp)	Read length N50	Reads depth	GC (Guanine-cytosine) content (%)
IEDCR9837394	01-08-2024	56	Male	Dhaka	Asymptomatic	PP301385	EPI_ISL_18787954	SRR27909866	314,310	29,721	511	452x	39.67
IEDCR1802057	12-30-2023	12	Male	Gazipur	Asymptomatic	PP301386	EPI_ISL_18787955	SRR27909867	430,999		515	449x	39.71
IEDCR9848794	12-19-2023	58	Male	Dhaka	Asymptomatic	PP301387	EPI_ISL_18787957	SRR27909868	90,263		509	344x	39.26
IEDCR1802649	12-30-2023	18	Male	Gazipur	Asymptomatic	PP301388	EPI_ISL_18787958	SRR27909869	257,355		503	443x	39.14
IEDCR9850602	01-12-2024	52	Female	Dhaka	Asymptomatic	PP301389	EPI_ISL_18851597	SRR27909870	302,710		515	452x	39.55
IEDCR9850683	01-12-2024	57	Male	Dhaka	Asymptomatic	PP301390	EPI_ISL_18851598	SRR27909871	126,879		516	317x	41.25
IEDCR2201113	01-13-2024	50	Female	Dhaka	Mild to moderate	PP301391	EPI_ISL_18851599	SRR27909872	358,693		507	454x	40.69
IEDCR1625069	01-14-2024	49	Female	Dhaka	Mild to moderate	PP301392	EPI_ISL_18851600	SRR27909873	128,654		515	371x	42.58
IEDCR1625075	01-14-2024	64	Male	Dhaka	Mild to moderate	PP301393	EPI_ISL_18851601	SRR27909874	45,187		516	345x	40.25
IEDCR1450955	01-15-2024	24	Female	Dhaka	Asymptomatic	PP301394	EPI_ISL_18851602	SRR27909875	111,939		501	420x	39.12
IEDCR9848717	01-16-2024	65	Female	Chandpur	Asymptomatic	PP301395	EPI_ISL_18851603	SRR27909876	198,273		517	377x	39.56
IEDCR1450962	01-16-2024	62	Female	Dhaka	Mild to moderate	PP301396	EPI_ISL_18851604	SRR27909877	70,507		515	381x	40.14
IEDCROIS23049	01-21-2024	56	Male	Dhaka	Hospitalized	PP301397	EPI_ISL_18851605	SRR27909878	72,169		510	390x	41.20
IEDCROIS23050	12-21-2023	12	Male	Gazipur	Hospitalized	PP301398	EPI_ISL_18851606	SRR27909879	69,016		514	356x	39.65

^*a*^ GISAID: Global Initiative of Sharing All Influenza Data.

^*b*^ SRA: Sequence Reads Archive.

## Data Availability

The data from this study can be found in GenBank under the accession number PP301385 to PP301398. Sequences can also be accessed in the GISAID (www.gisaid.org) using the accession numbers EPI_ISL_18787954, EPI_ISL_18787955, EPI_ISL_18787957, EPI_ISL_18787958, and EPI_ISL_18851597 to EPI_ISL_18851606. The raw sequencing reads were submitted at the Sequence Read Archive (SRA) database under BioPorject PRJNA1074515 with the individual accession number starting from SRR27909866 to SRR27909879.
